# Essential requirement for polypyrimidine tract binding proteins 1 and 3 in the maturation and maintenance of mature B cells in mice

**DOI:** 10.1002/eji.202149257

**Published:** 2021-07-26

**Authors:** Elisa Monzón‐Casanova, Kirsty J. Bates, Christopher W. J. Smith, Martin Turner

**Affiliations:** ^1^ Laboratory of Lymphocyte Signalling and Development, The Babraham Institute Babraham Research Campus Cambridge UK; ^2^ Department of Biochemistry University of Cambridge Cambridge UK

**Keywords:** mature B cells, posttranscriptional gene expression regulation, PTBP1, PTBP3, RNA binding proteins

## Abstract

The maturation of immature B cells and the survival of mature B cells is stringently controlled to maintain a diverse repertoire of antibody specificities while avoiding self‐reactivity. At the molecular level this is regulated by signaling from membrane Ig and the BAFF‐receptor that sustain a pro‐survival program of gene expression. Whether and how posttranscriptional mechanisms contribute to B cell maturation and survival remains poorly understood. Here, we show that the polypyrimidine tract binding proteins (PTBP) PTBP1 and PTBP3 bind to a large and overlapping set of transcripts in B cells. Both PTBP1 and PTBP3 bind to introns and exons where they are predicted to regulate alternative splicing. Moreover, they also show high‐density of binding to 3’ untranslated regions suggesting they influence the transcriptome in diverse ways. We show that PTBP1 and PTBP3 are required in B cells beyond the immature cell stage to sustain transitional B cells and the B1, marginal zone and follicular B cell lineages. Therefore, PTBP1 and PTBP3 promote the maturation of quiescent B cells by regulating gene expression at the posttranscriptional level.

## Graphical abstract

The RNA binding proteins PTBP1 and PTBP3 are expressed in B cells. We show their fundamental roles in promoting the maturation and maintenance of B cells. PTBP1 and PTBP3 bind to numerous transcripts and thereby promote B cell maintenance and maturation by controlling gene expression at the posttranscriptional level.

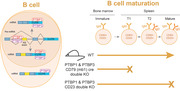

## Introduction

The maturation of naïve B cells occurs through discrete stages. In the bone marrow, developing B cells express IgM at their cell surface and are defined as immature. These B cells migrate to the spleen where they mature through different transitional stages (T1 and T2) [1, 2], express BAFF‐receptor and establish a homeostatic survival‐circuit dependent upon its ligand [3, 4]. Mature naïve B cells express IgD in addition to IgM and may recirculate between lymphoid follicles or reside in the marginal zones of the spleen. The maturation and survival of mature B cells is dependent upon signaling by surface Ig, and gene regulation by DNA binding transcription factors [4]. The contribution of posttranscriptional regulation of mRNA processing, stability, or translation is less well characterized [5, 6].

RNA binding proteins (RBP) acting posttranscriptionally determine the quantity and variants of transcripts produced from genes. They also regulate where, and in what amounts, mRNAs are translated. The role of these regulatory processes in B cell development is a new and exciting area. Polypyrimidine tract binding protein (PTBP) 1 is a widely expressed alternative splicing regulator. It has additional, less well‐characterized, roles in regulating microRNA processing, mRNA abundance, alternative polyadenylation, and internal ribosome entry site‐mediated translation [7]. Studies of B cells with *Cd79a(Mb1)^Cre^*‐mediated conditional deletion of *Ptbp1* showed it was dispensable for B cell development, but important for the generation of GC B cells and antigen‐specific antibodies [8, 9]. Absence of PTBP1 in B cells led to the expression of PTBP2 [8], which is expressed in the brain [7]. In mice with *Cd79a* *^Cre^*‐mediated conditional deletion of both *Ptbp1* and *Ptbp2*, B cell development is blocked at the pro‐B cell stage [10]. This is associated with defective cell cycle regulation in pro‐B cells and shows that PTBP2, which is silenced by PTBP1, compensates for the lack of PTBP1.

The paralog PTBP3 is expressed specifically in cells of the hematopoietic system. In B cells, PTBP1 and PTBP3 are found in the nucleus whereas PTBP3 is also detected in the cytoplasm [11]. The prominent cytoplasmic localization of PTBP3 suggests that in addition to nuclear roles it may also affect mRNA stability, translation, or localization. Knowledge of PTBP3 in physiological systems is limited with a report that *Ptbp3* ^–/–^ mice have normal B cell development, but defective antibody responses [12]. However, when *Ptbp3* was deleted conditionally in B cells with the *Cd79a^Cre^* allele we found normal B cell development, antibody amounts, and affinity maturation in response to immunization ([10] and unpublished data). Here, we show that PTBP1 and PTBP3 act redundantly to promote the maturation and maintenance of B cells.

## Results and discussion

### PTBP1 and PTBP3 bind redundantly to introns and 3’‐UTRs

**Figure 1:**
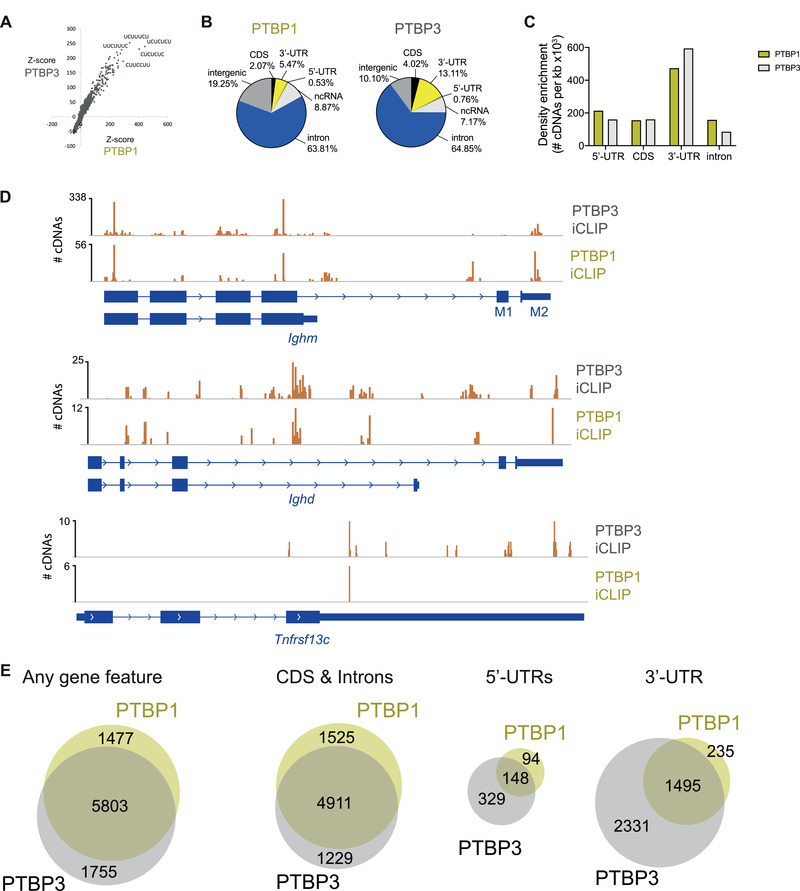
PTBP1 and PTBP3 bind redundantly to the transcriptome of B cells. (A) *Z*‐score correlation of motifs found in PTBP1 and PTBP3 iCLIPs. (B) Proportions of xlink‐binding sites in different genomic features in PTBP1 and PTBP3 iCLIPs. (C) Density enrichment of xlink‐binding sites in different genomic features in PTBP1 and PTBP3 iCLIPs. (D) Significant (FDR<0.05) xlink‐binding sites and number of cDNAs identified on each site along *Ighm, Ighd*, and *Tnfrsf13c* transcripts in PTBP1 and PTBP3 iCLIPs. (E) Overlaps of number of genes with a xlink‐binding site (FDR < 0.05) with a height ≥3 meaning that the xlink‐binding site was identified with ≥3 independent cDNAs in PTBP1 and PTBP3 iCLIPs.

Since both PTBP1 and PTBP3 are highly homologous, particularly within their RNA binding domains, we compared their transcript‐binding profile by direct mapping binding sites in the B cell transcriptome using individual‐nucleotide resolution UV cross‐linking and immunoprecipitation (iCLIP) [13]. We used LPS‐activated splenic B cells. A comparison of the genes expressed with at least one FPKM in LPS‐activated B cells to *ex vivo*‐isolated splenic B cells revealed a 91% overlap (Supporting Information Fig. S1A). These iCLIPs revealed the same polypyrimidine‐tract binding motifs for PTBP1 and PTBP3 (Fig. 1A). In addition to the expected binding to introns, we found prominent binding of both proteins to 3’ untranslated regions (3’‐UTRs) (Fig. 1B). We found a higher density of PTBP3 binding to 3’‐UTRs compared to PTBP1 (Fig. 1C). Conversely, there was a higher density of PTBP1 binding to introns compared to PTBP3 (Fig. 1C), which is consistent with the predominant nuclear localisation of PTBP1 [11].

Detailed analysis of individual binding sites, such as those in transcripts encoding IgM and IgD heavy chains (*Ighm* and *Ighd*) and the BAFF‐receptor (*Tnfrsf13c*), showed that PTBP1 and PTBP3 bound largely to the same sites (Fig. 1D). We observed binding of both PTBPs to the IgM M2 exon where PTBP1 has been shown to suppress an enhancer of splicing that promoted exon inclusion [14]. To define reproducible binding sites, we chose a cut‐off of at least three independent cDNA reads in the iCLIP (and FDR<0.05). With these settings, we found that PTBP1 and PTBP3 bound to transcripts of 9,035 genes with a large overlap of 64% (5,803 genes) (Fig. 1E, Supporting Information Table S1). This is an underestimate of genes bound by both PTBPs, since there will be genes bound by both PTBPs for which one PTBP did not pass our cut‐off criteria. PTBP3 bound to the 5’‐UTR and 3’‐UTR of more genes than PTBP1 (Fig. 1E), reflecting the greater cytoplasmic localization of PTBP3 compared to PTBP1. Amongst the 12,657 polyadenylated genes detected in LPS‐activated B cells with at least one FPKM, PTBP1 and PTBP3 bound to 7,644 (60%) (Supporting Information Fig. S1B), highlighting their potential to regulate numerous genes and pathways. Altogether, although we found some small differences, these RBPs showed a highly redundant pattern of binding consistent with them regulating the same RNAs in B cells.

### PTBP1 and PTBP3 are necessary for B cell maturation

**Figure 2:**
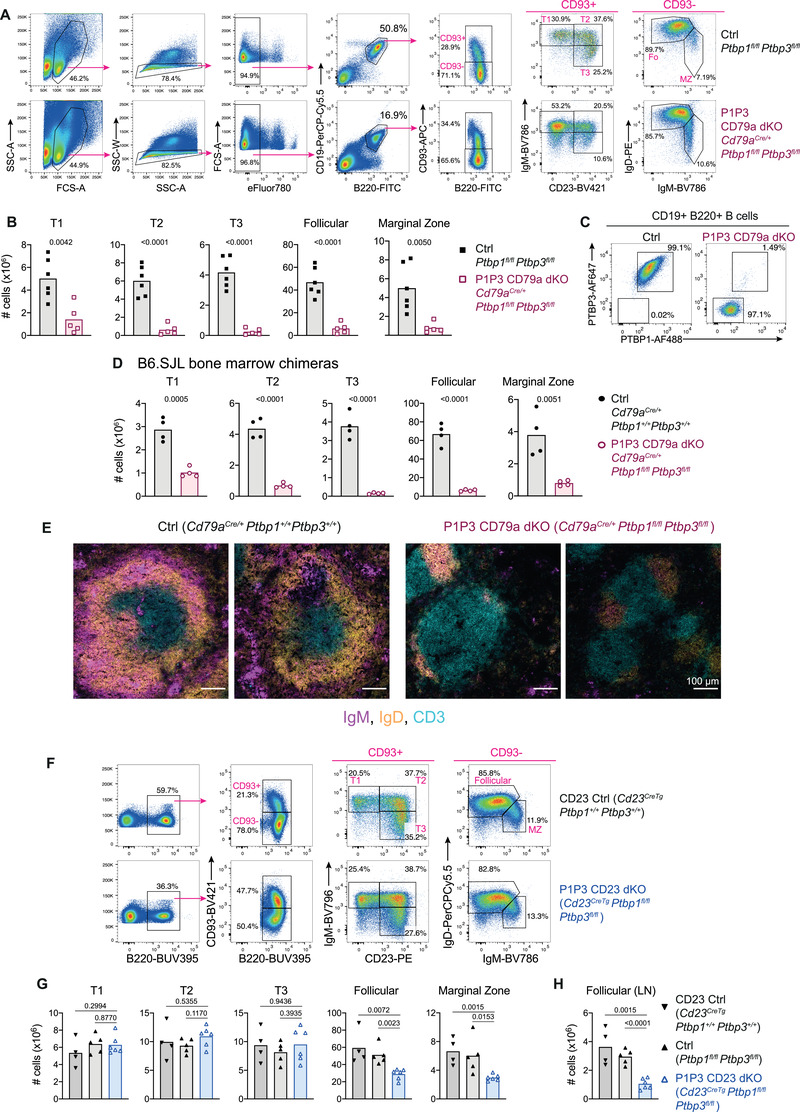
PTBP1 and PTBP3 are necessary for B cell maturation and maintenance. (A) Flow cytometry gating strategy identifying B cell populations amongst splenocytes. Numbers shown are percentages. (B) Numbers of cells in spleens identified as shown in (A). Bars show arithmetic means. Each symbol shows data from one mouse. *P*‐values from two‐tailed unpaired Student's *t*‐test are shown. Data from five‐ to six‐mice pooled from two independent experiments are shown. (C) Intracellular PTBP1 and PTBP3 staining analysed by flow cytometry in splenic B cells. Full gating strategy is shown in Supporting Information Fig. S2B. Data shown are from one mouse of each genotype from one experiment with three mice per genotype. (D) Numbers of cells in spleens from B6.SJL mice irradiated and reconstituted with bone marrow cells from control (*Cd79a^Cre/+^Ptbp1^+/+^Ptbp3^+/+^*) or P1P3 CD79a dKO mice. Flow cytometry gating strategy is shown in Supporting Information Fig. S2D. Each symbol shows data from one mouse. *P*‐values shown are from a two‐tailed unpaired Student's *t*‐test. Bars show arithmetic means. Data are from one experiment with four mice per group. (E) Immunofluorescence of spleen sections from B6.SJL mice reconstituted with bone marrow cells from control (*Cd79a^Cre/+^Ptbp1^+/+^Ptbp3^+/+^*) or P1P3 CD79a dKO mice. B cells were identified with anti‐IgM shown in magenta and anti‐IgD shown in yellow. T cells were identified with anti‐CD3 shown in blue. Images were captured at 20× magnification. Each image is from a different mouse. Data are from one experiment with four mice. (F) Flow cytometry gating strategy to identify B cell populations among splenocytes. Events shown in first pseudocolor plots have been pre‐gated on live (eFluor780‐) single‐cell lymphocytes. (G) Numbers of cells in spleens identified as shown in F. Bars show arithmetic means. Each symbol shows data from one mouse. *P*‐values from two‐tailed unpaired Student's *t*‐test are shown. Data shown are representative from one out of two independent experiments with two to six mice per genotype in each experiment. (H) Numbers of follicular B cells found in peripheral (axillar, inguinal, and brachial) lymph nodes. Each symbol shows data from one mouse. *P*‐values from two‐tailed unpaired Student's *t*‐test are shown. Data shown are from one representative out of two independent experiments with two to six mice per genotype per experiment. Gating strategy is shown in Supporting Information Fig. S3B.

We found previously that *Cd79a^Cre^*‐mediated deletion of *Ptbp1* and *Ptbp3* together did not affect the numbers of pre‐ and immature‐B cells in the bone marrow in *Cd79a^Cre/+^Ptbp1^fl/fl^Ptbp3^fl/fl^* (P1P3 CD79a dKO) mice [10]. In contrast, in the spleen, we observed a reduction in the frequency of B220+CD19+ cells (Fig. 2A). Among CD93^+^ B cells in P1P3 CD79a dKO mice, there was an increase in the proportions of the first transitional T1 cells compared to littermate control mice (Fig. 2A). The enumeration of cells showed an almost complete absence of mature B cells in P1P3 CD79a dKO mice (Fig. 2B). The numbers of cells in the T2 and T3 subsets were reduced by 9.7‐ and 17‐fold, respectively, with the T1 subset the least affected being reduced by 3.5‐fold. In the peritoneal lavage, there were few B cells and CD19^high^B220^low^ B1 B cells were clearly depleted (Supporting Information Fig. S2A). Intracellular staining for PTBP1 and PTBP3 in B cells from P1P3 CD79a dKO mice showed efficient protein depletion but, unlike control cells, they express PTBP2 (Fig. 2C; Supporting Information Fig. S2B).

We found that P1P3 CD79a dKO mice became severely unwell and had to be culled between 10–12 weeks of age. At necropsy, it was apparent that the kidneys were abnormal with signs of polycystic lesions (Supporting Information Fig. S2C). There have been reports of *Cd79a^Cre^* expression in the kidney [15] and conditional KO of *Tsc1* with *Cd79a^Cre^* led to a polycystic kidney phenotype [16], suggesting that *Ptbp1* and *Ptbp3* deletion with the *Cd79a^Cre^* led to these lesions, but this will have to be confirmed. To establish if the B cell phenotype was intrinsic to the hematopoietic system we transferred bone marrow from *Cd79a^Cre/+^Ptbp1^+/+^Ptbp3^+/+^* control or P1P3 CD79a dKO mice into lethally irradiated B6.SJL mice. There was no early‐onset disease or kidney abnormalities found in these chimeras. Following reconstitution, as with intact mice, the numbers of all splenic transitional and mature B cell subsets in P1P3 CD79a dKO were substantially reduced compared to control chimeras (Fig. 2D; Supporting Information Fig. S2D). The proportions of B cells and the ratio of B cells compared to T cells in the blood were reduced 2.4‐ and 5.7‐fold, respectively, in P1P3 CD79a dKO compared to control chimeras (Supporting Information Fig. S2E and S2F). Histological examination of the spleens from P1P3 CD79a dKO chimeras showed the few B cells present localized around the T cell zone indicating unaffected migration of the PTBP1 and PTBP3‐deficient B cells in the spleen (Fig. 2E). A defect in the export of PTBP1 and PTBP3 deficient immature B cells could contribute to the reduced number of T1 B cells. These data revealed the indispensable role of PTBP1 and PTBP3 in the differentiation or maintenance of mature B cells in the spleen and the peritoneum—a role that cannot be compensated for by PTBP2.

### PTBP1 and PTBP3 are necessary for the maintenance of mature B cells

To study the mature B cell compartment further we used *Cd23^CreTg^* [17], which mediates recombination in transitional B cells to generate *Cd23^CreTg^Ptbp1^fl/fl^Ptbp3^fl/fl^* mice (P1P3 CD23 dKO) and analyzed splenic B cell subsets by flow cytometry (Fig. 2F). The numbers of T1, T2, and T3 B cells in P1P3 CD23 dKO mice were similar to those in *Cd23^CreTg^Ptbp1^+/+^Ptbp3^+/+^* or *Ptbp1^fl/fl^Ptbp3^fl/fl^* mice (Fig. 2G). By contrast, the numbers of B cells with the follicular or marginal zone (MZ) phenotype in the spleen were substantially reduced (Fig. 2G). Identification of MZ B cells with staining of CD21 and CD23 also showed a reduction in MZ B cell numbers in the spleen and revealed lower staining of CD21 in PTBP1 and PTBP3‐deficient cells compared to control B cells (Supporting Information Fig. S3A). In addition, the numbers of B cells from lymph nodes were reduced by three‐fold in P1P3 CD23 dKO mice (Fig. 2H, Supporting Information Fig. S3B).

The presence of both PTBP1 and PTBP3 proteins in T1 and T2 cells from P1P3 CD23 dKO mice (Supporting Information Fig. S3C) was consistent with the minimal impact on cell numbers at these stages. Follicular B cells in both the spleen and lymph node showed a large proportion of cells that retained expression of PTBP1 and PTBP3 (Supporting Information Fig. S3C and S3D) at the levels of control and *Ptbp1* and *Ptbp3* heterozygous CD23 KO mice (Supporting Information Fig. S3E). Yet their absolute numbers were reduced two to three‐fold in the P1P3 CD23 dKO mice (Fig. 2G and H). Moreover, cells with maintained PTBP1 and PTBP3 expression were abundant amongst MZ B cells (Supporting Information Fig. S3C).

We assessed IgM and IgD surface levels in PTBP1 and PTBP3‐deficient mature B cells from P1P3 CD23 dKO mice where we could assess consequences of recent PTBP1 and PTBP3 protein depletion in B cells. IgM and IgD staining in PTBP1 and PTBP3‐deficient mature B cells were unaffected (Supporting Information Fig. S3F). Also, BTK and AKT phosphorylation at Tyrosine 223 and Threonine 308, respectively, upon IgM crosslinking was unaffected in PTBP1 and PTBP3‐deficient mature B cells (data not shown). PTBP1 and PTBP3‐deficient mature B cells expressed PTBP2 (Supporting Information Fig. S3C) suggesting that PTBP2 compensated for some roles of PTBP1 and PTBP3. Nonetheless, PTBP2 in follicular and MZ B cells was unable to support these cells revealing a role for PTBP1 and PTBP3 in the maintenance of mature B cell subsets.

### Reduced BAFF‐receptor in the absence of PTBP1 and PTBP3

**Figure 3:**
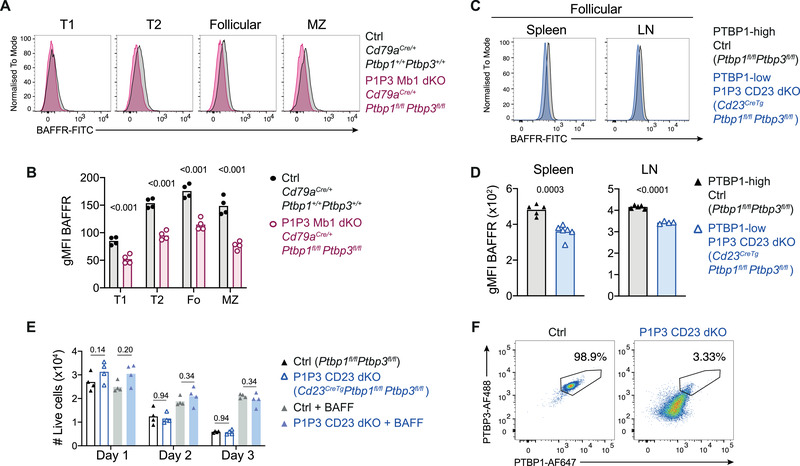
Reduced BAFF‐receptor in the absence of PTBP1 and PTBP3. (A) BAFF‐receptor (BAFFR) staining in splenic B cells from B6.SJL chimeric mice and measured by flow cytometry. B cell populations were identified as shown in Supporting information Fig. S2D. (B) Geometric mean fluorescence intensity (gMFI) of the staining shown in A. (C) BAFFR staining of follicular B cells from the spleen and lymph nodes and measured by flow cytometry. B cells were pre‐gated on the levels of PTBP1 as shown in Supporting Information Fig. S3F. (D) gMFI of the staining shown in C. (E) Live B cells isolated from lymph nodes recovered after several days of culture with or without exogenous BAFF (200 ng/ml). Live cells (eFluor780‐) were analyzed by flow cytometry. (F) Intracellular staining of live B cells (eFluor780‐, B220+) cultured for one day without BAFF as shown in E. Intracellular staining of B cells cultured with BAFF was similar to that without BAFF. In (B, D, and E), each point shows data from one mouse. Bars show arithmetic means. In (B and E) *P*‐adjusted values from two‐tailed Student's *t*‐test corrected by two‐stage step‐up method of Benjamini, Krieger, and Yekutieli are shown. (D) *P*‐values from unpaired two‐tailed Student's *t*‐test are shown. In (B and D), data shown are from one experiment with four to six mice per genotype. (E) Data shown are from one of two independent experiments with three to four mice per genotype in each experiment.

BAFF signaling is critical for the maturation and maintenance of B cells [3, 4] and BAFF‐receptor is induced by tonic signaling [18]. PTBP1 and PTBP3 bound to the 3’UTR of *Tnfrsf13c* (encoding BAFF‐receptor) (Fig. 1D). BAFF‐receptor staining was reduced in PTBP1 and PTBP3 deficient B cells from the P1P3 CD79a dKO chimeras and the P1P3 CD23 dKO mice (Fig. 3A–D). This reduction could be a direct effect of PTBP1 and PTBP3 in controlling *Tnfrsf13c* mRNA abundance or translation or also a result of impaired tonic signaling. We cultured B cells isolated from lymph nodes of P1P3 CD23 dKO mice and control littermate mice *ex vivo* with or without exogenous BAFF (Fig. 3E). The numbers of B cells from P1P3 CD23 dKO mice we recovered after several days in culture were unaffected even though B cells from P1P3 CD23 dKO in culture had lost PTBP1 and PTBP3 proteins (Fig. 3F). This showed that PTBP1 and PTBP3‐deficient mature B cells respond to exogenous BAFF which promotes their survival *ex vivo*. Thus, we expect that in vivo PTBP1 and PTBP3 are necessary for controlling expression at the posttranscriptional level of many other genes necessary for B cell maturation and maintenance in addition to BAFF‐receptor and the absence of B1 cells in P1P3 CD79 dKO mice reflects this.

## Concluding remarks

The requirement of both PTBP1 and PTBP3 in transitional and mature B cells contrasts with early B cell development where PTBP2 compensated completely for the absence of these proteins [10]. In mouse brain, PTBP1 and PTBP2 bind redundantly largely to the same sites [19]. Therefore, we expect PTBP2 to bind to the same sites as PTBP1 and PTBP3 do in B cells. The inability of PTBP2 to sustain B cell maturation may reflect qualitative differences between the paralogs, as PTBP2 is not thought to enter the cytoplasm. However, it is also possible that all three paralogs have redundant roles and that PTBP2 abundance is insufficient to ensure B cell maturation and maintenance.

Given their widespread binding, we expect that PTBP1 and PTBP3 will control many genes necessary for the maintenance of mature B cell populations. In addition to *Tnfrsf13c* binding, PTBP1 and PTBP3 bound to exons, introns, and 3’UTRs *Bcl2*, *Syk*, *Akt1, Cxcr4*, and *S1pr1* among many other genes (Supporting Information Table S1 and Fig. S1B). Further investigations are warranted to elucidate if and how PTBP1 and PTBP3 control expression of these targets necessary for the export of immature B cells from the bone marrow and survival, tonic signaling, and maintenance of mature B cells. We anticipate diverse and shared roles for PTBP1 and PTBP3 in B cells including the control of alternative splicing and also other functions via 3’‐UTR‐binding such as mRNA localization, stability, and translation.

## Materials and methods

### Mice and bone marrow chimeras

Mice were maintained in the Babraham Institute Biological Support Unit. No primary pathogens or additional agents listed in the FELASA recommendations have been confirmed during health monitoring since 2009. Ambient temperature was ∼19–21°C and relative humidity 52%. Lighting was provided on a 12hr light: 12hr dark cycle including 15 min ‘dawn’ and ‘dusk’ periods of subdued lighting. After weaning, mice were transferred to individually ventilated cages with one to five mice per cage. Mice were fed CRM (P) VP diet (Special Diet Services) ad libitum and received seeds (e.g., sunflower, millet) at the time of cage‐cleaning as part of their environmental enrichment. All mouse experimentation was approved by the Babraham Institute Animal Welfare and Ethical Review Body. Animal husbandry and experimentation complied with existing European Union and United Kingdom Home Office legislation. All mice were used experimentally between 8‐ and 25‐weeks of age (for bone marrow chimeras) and were age‐ and sex‐matched within experiments. No sex‐associated differences were observed in the results obtained. Mice were on the C57BL/6 background and derived from crossing the following transgenic strains: *Ptbp1^fl/fl^* (Ptbp1^tm1Msol^) [20], *Ptbp3^fl/fl^* (Ptbp3^tm1Tnr^) [10], *Cd79a^Cre^* (Cd79^atm1(cre)Reth^) [15], and *Cd23^CreTg^* (Cd23‐cre^Tg(Fcer2a‐cre)5Mbu^) [17]. To generate chimeras, B6.SJL mice were lethally irradiated (2 × 0.5 Gy) and reconstituted with 3 × 10^6^ bone marrow cells derived from control (*Cd79a^Cre/+^Ptbp1^+/+^Ptbp3^+/+^*) or P1P3 CD79a dKO mice.

### Flow cytometry

Cell surface and intracellular staining for flow cytometry analyses were carried out as described [10] adhering to the guidelines for the use of flow cytometry [21]. The antibodies used were: B220‐FITC (RA3‐6B2, Tonbo, cat#:35‐0452), B220‐PECy7 (RA3‐6B2, eBioscience, cat#:25‐0452‐82), B220‐BUV395 (RA3‐6B2, BD, cat#:563793), CD19‐PerCPCy5.5 (1D3, Biolegend, cat#:152406), CD19‐BUV395 (1D3, BD, cat#:565965), CD19‐BUV737 (1D3, BD, cat#:564296), CD19‐BV421 (6D5, Biolegend, cat#:115549), CD93‐APC (AA4.1, eBioscience, cat#:17‐5892‐83), CD93‐BV421 (AA4.1, BD, cat#: 747716), CD23‐BV421 (B3B4, Biolegend, cat#:101621), CD23‐PE (B3B4, BD, cat#: 553139), IgM‐BV786 (R6‐60.2, BD, cat#: 564028), IgM‐PECy7 (II/41, eBioscience, cat#: 25‐5790‐81), IgD‐PE (11‐26, SouthernBiotech, cat#:1120‐09L), IgD‐BV510 (11‐26c.2a, Biolegend, cat#:405723), IgD‐PerCPCy55 (11‐26c.2a, Biolegend cat#:405710), TCRb‐BUV737 (H57‐597, BD cat#:564799), CD45.2‐BV785 (104, Biolegend, cat#:109839), BAFFR‐FITC (eBio7H22.E16, eBioscience, cat# 11‐5943‐82). Anti‐PTBP1 (CLONE 1, ThermoFisher, cat#:32‐4800), anti‐PTBP2 (S43, from Michele Solimena), and anti‐PTBP3 (MAC454, [8]) antibodies were conjugated to different fluorophores with antibody labeling kits from ThermoFisher (AF488, cat#:A20181; AF647, cat#:A20186 and AF594, cat#:A20185). eFluor780 (ThermoFisher, cat#: 65‐0865‐18) cell surface staining was used to identify live cells. Data were analyzed with Flowjo (v 10.7.1). Statistical analyses were carried out with Prism 8 (v 8.4.3).

### B cell isolation and ex vivo culture

B cells were isolated from the lymph nodes using the mouse B cell isolation kit (Miltenyi, cat#130‐090‐862). 10^5^ B cells were cultured per well in 96 flat‐bottom well plates in RPMI 1640 Medium (Dutch modification, ThermoFisher, cat#:22409‐015) supplemented with Glutamax, 10% FCS, 50 μM β‐mercaptoethanol, and 100 units/ml Penicillin and Streptomycin for several days in the presence or absence of 200 ng/ml exogenous human BAFF (PeproTech, cat#: 310–13).

### Immunofluorescence

Spleens from chimeras were embedded in optimal temperature cutting medium (OCT) and 10 μm sections cut using a Leica cryostat. Sections were fixed in acetone at −20°C, blocked with 2% BSA and 10% goat serum, and permeabilized in 2% Triton‐X. Sections were stained with rat anti‐mouse IgD‐AF647 (11–26c.2a, BioLegend, cat#:405708); rat anti‐mouse IgM‐BV421 (R6‐60.2, BD, cat#:562595); and unconjugated hamster anti‐mouse CD3 (500A2, eBioscience, cat#:14‐0033‐85) followed by goat anti‐hamster IgG‐AF568 (ThermoFisher, cat#:A‐21112). Confocal images were acquired with a Nikon A1‐R confocal microscope using the 20x/0.8 air objective lens. Laser excitation/emission detection bands for the three different fluorescent dyes were as follows (all in nm): BV421, ex408 em450/50; Alexa Fluor 568, ex562 em595/50, and Alexa Fluor 647, ex640 em700/75. The system was controlled using Nikon Elements software, with each fluorescence channel acquired independently to minimize spectral bleed through. ImageJ software was used to process the images.

### iCLIP

The PTBP3 iCLIP reported in this study was carried out with B cells isolated from the spleen stimulated ex vivo for 48h with LPS (10 μg/ml, 127:B0, Sigma) as described in [8]. We used 10 μg of the anti‐PTBP3 rabbit polyclonal antibody PTBP3‐L2‐3 [11] and 100 μl protein A magnetic Dynabeads (ThermoFisher, cat#:10002D). Details of the barcodes and oligos used can be accessed in GEO under: GSE168769. iCLIP libraries were mapped to the GRCm38 mouse genome M16 release version from Gencode with STAR [22]. cDNA reads were de‐duplicated using random barcodes included in the library preparation and significant (FDR<0.05) xlink‐binding sites were identified with iCount (https://icount.readthedocs.io/en/latest/cite.html). PTBP1 iCLIP was published previously [8, 10]. FPKMs of ex vivo isolated and LPS‐activated splenic B cells were calculated from mRNAseq data published under the GSE62129 accession number [23].

## Author contributions

E.M.C., C.W.J.S., and M.T. conceptualized the study; E.M.C and K.J.B. carried out experiments; E.M.C. analyzed data; E.M.C and M.T. wrote the manuscript with input from all the authors. E.M.C., C.W.J.S., and M.T. acquired funding for the study.

## Conflicts of interest

The authors declare no commercial or financial conflicts of interest.

### Peer review

The peer review history for this article is available at https://publons.com/publon/10.1002/eji.202149257

## Supporting information

Supporting Information

Table S1

## Data Availability

The data that support the findings of this study are openly available in GEO at https://www.ncbi.nlm.nih.gov/geo/, reference number GSE136882 for PTBP1 and reference number GSE168769 for PTBP3 iCLIPs.
