# Supplementary material for: Essential requirement for polypyrimidine tract binding proteins 1 and 3 in the maturation and maintenance of mature B cells in mice
Source: Eur J Immunol. 2021 Jul 26;51(9):2266–73. doi: 10.1002/eji.202149257 (PMC11146436; doi:10.1002/eji.202149257)
Supplement: Supplementary file 1 — Supporting Information [file EJI-51-2266-s001.pdf]

Supporting Information *Monzón-Casanova et al.*

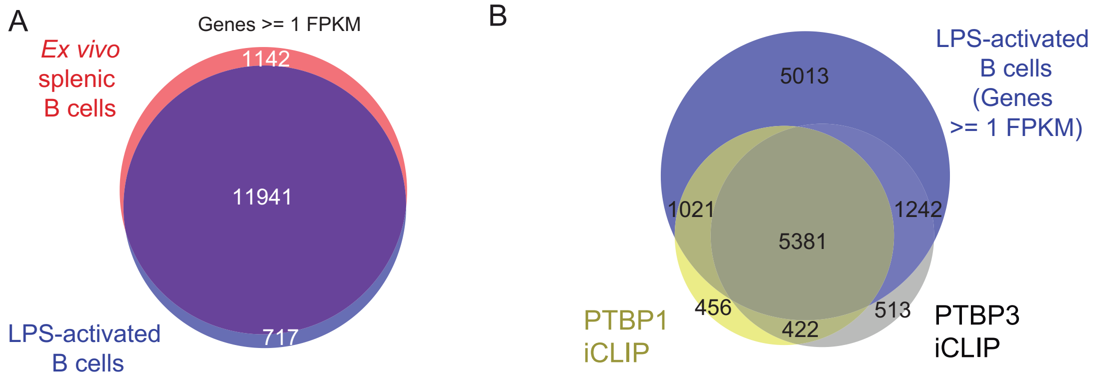

Figure S1

Figure S1. Overlap of genes expressed in B cells with genes whose transcripts are bound by PTBP1 and PTBP3.
(A) Overlap of genes expressed with >= 1 FPKM (arithmetic mean of 4 independent replicates) in *ex vivo* isolated splenic B cells and splenic B cells activated with LPS for 48h [23].
(B) Overlap of genes expressed in LPS-activated B cells (with >= 1 FPKM) and those whose transcripts are bound by PTBP1 and/or PTBP3.

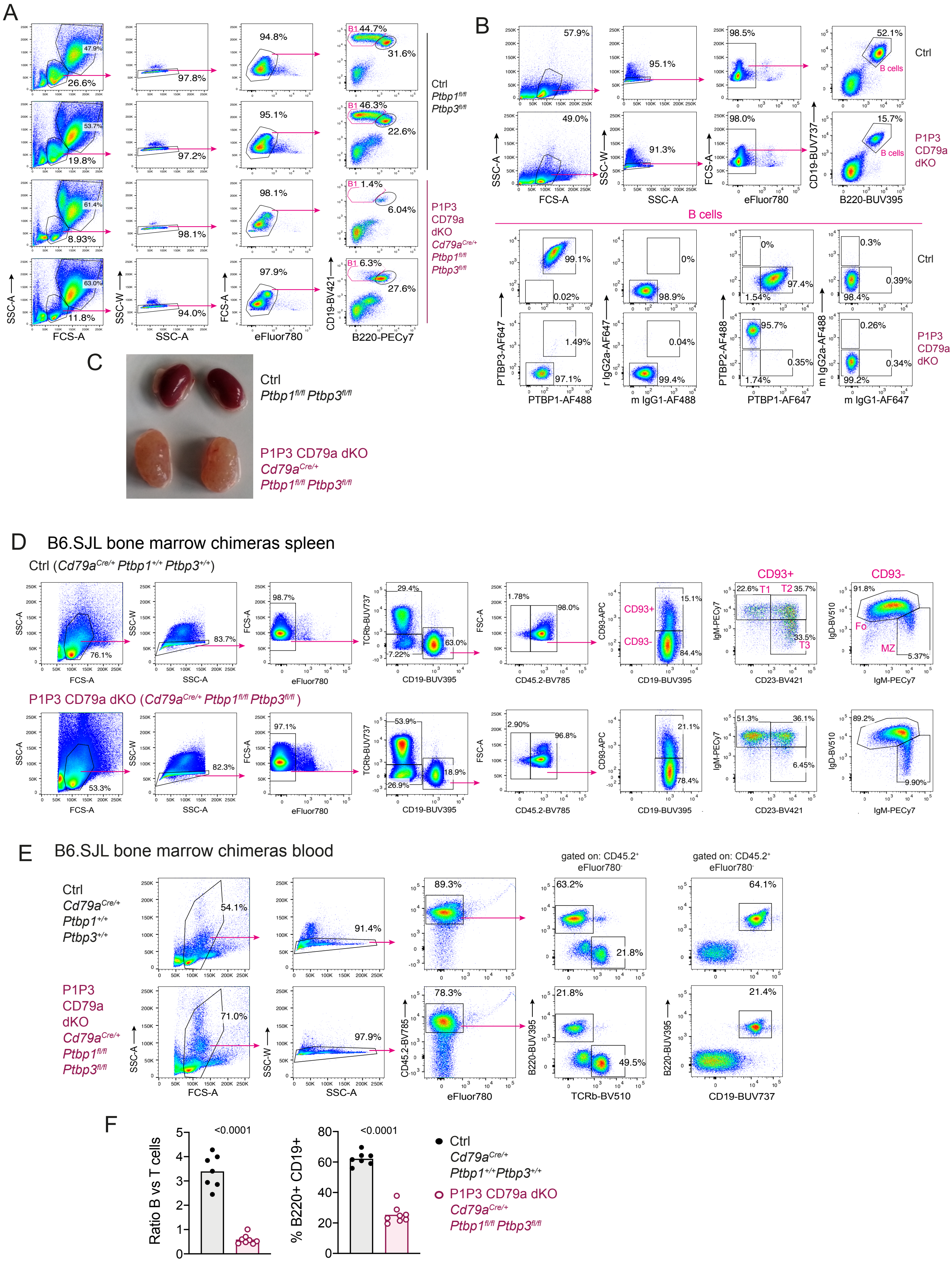
A
Ctrl
Ptbp1fl/fl
Ptbp3fl/fl
P1P3
CD79a
dKO
Cd79aCre/+
Ptbp1fl/fl
Ptbp3fl/fl
SSC-A
FCS-A
SSC-W
SSC-A
FCS-A
eFluor780
CD19-BV421
B220-PECy7
B
Ctrl
P1P3
CD79a
dKO
B cells
SSC-A
FCS-A
SSC-W
SSC-A
FCS-A
eFluor780
CD19-BUV737
B220-BUV395
PTBP3-AF647
PTBP1-AF488
r IgG2a-AF647
m IgG1-AF488
PTBP2-AF488
PTBP1-AF647
m IgG2a-AF488
m IgG1-AF647
C
Ctrl
Ptbp1fl/fl Ptbp3fl/fl
P1P3 CD79a dKO
Cd79aCre/+
Ptbp1fl/fl Ptbp3fl/fl
D
B6.SJL bone marrow chimeras spleen
Ctrl (Cd79aCre/+ Ptbp1+/+ Ptbp3+/+)
P1P3 CD79a dKO (Cd79aCre/+ Ptbp1fl/fl Ptbp3fl/fl)
CD93+
CD93-
T1
T2
T3
Fo
MZ
FCS-A
SSC-A
eFluor780
CD19-BUV395
CD45.2-BV785
CD23-BV421
IgM-PECy7
TCRb-BUV737
CD93-APC
IgD-BV510
E
B6.SJL bone marrow chimeras blood
gated on: CD45.2+ eFluor780-
Ctrl
Cd79aCre/+
Ptbp1+/+
Ptbp3+/+
P1P3
CD79a
dKO
Cd79aCre/+
Ptbp1fl/fl
Ptbp3fl/fl
FCS-A
SSC-A
eFluor780
TCRb-BV510
CD19-BUV737
CD45.2-BV785
B220-BUV395
F
Ratio B vs T cells
% B220+ CD19+
<0.0001
Ctrl
Cd79aCre/+
Ptbp1+/+Ptbp3+/+
P1P3 CD79a dKO
Cd79aCre/+
Ptbp1fl/fl Ptbp3fl/fl

Figure S2

Figure S2. PTBP1 and PTBP3 are necessary for B cell maturation.
(A) Flow cytometry analysis and gating strategy of B1 cells in the peritoneum of mice with the indicated genotypes. Data shown are from two mice per genotype.
(B) Flow cytometry analysis of PTBP1, PTBP2 and PTBP3 intracellular staining in splenic B cells from control and P1P3 CD79a dKO mice. Top two rows show gating strategy of B cells. Pseudocolour plots in the bottom two rows show intracellular staining for the different PTBPs and their respective control staining using matching antibody isotypes. Data are representative of one out of three mice per genotype.
(C) Kidneys from representative mice. Polycystic kidneys were found in P1P3 CD79a dKO mice with 100% prevalence amongst 30 mice examined.
(D) Flow cytometry gating strategy to identify B cell populations amongst splenocytes of B6.SJL mice reconstituted with bone marrow cells from control ($Cd79a^{Cre/+}Ptbp1^{+/+}Ptbp3^{+/+}$) or P1P3 CD79a KO mice.
(E) Flow cytometry analysis and gating strategy of cells from the blood of B6.SJL mice reconstituted with bone marrow cells from control or P1P3 CD79a KO mice.
(F) Data from flow cytometry analysis shown in E. Bars show arithmetic means, each data point shows data from one mouse. P values from two-tailed unpaired Student's t-test are shown. Data shown is from one experiment.

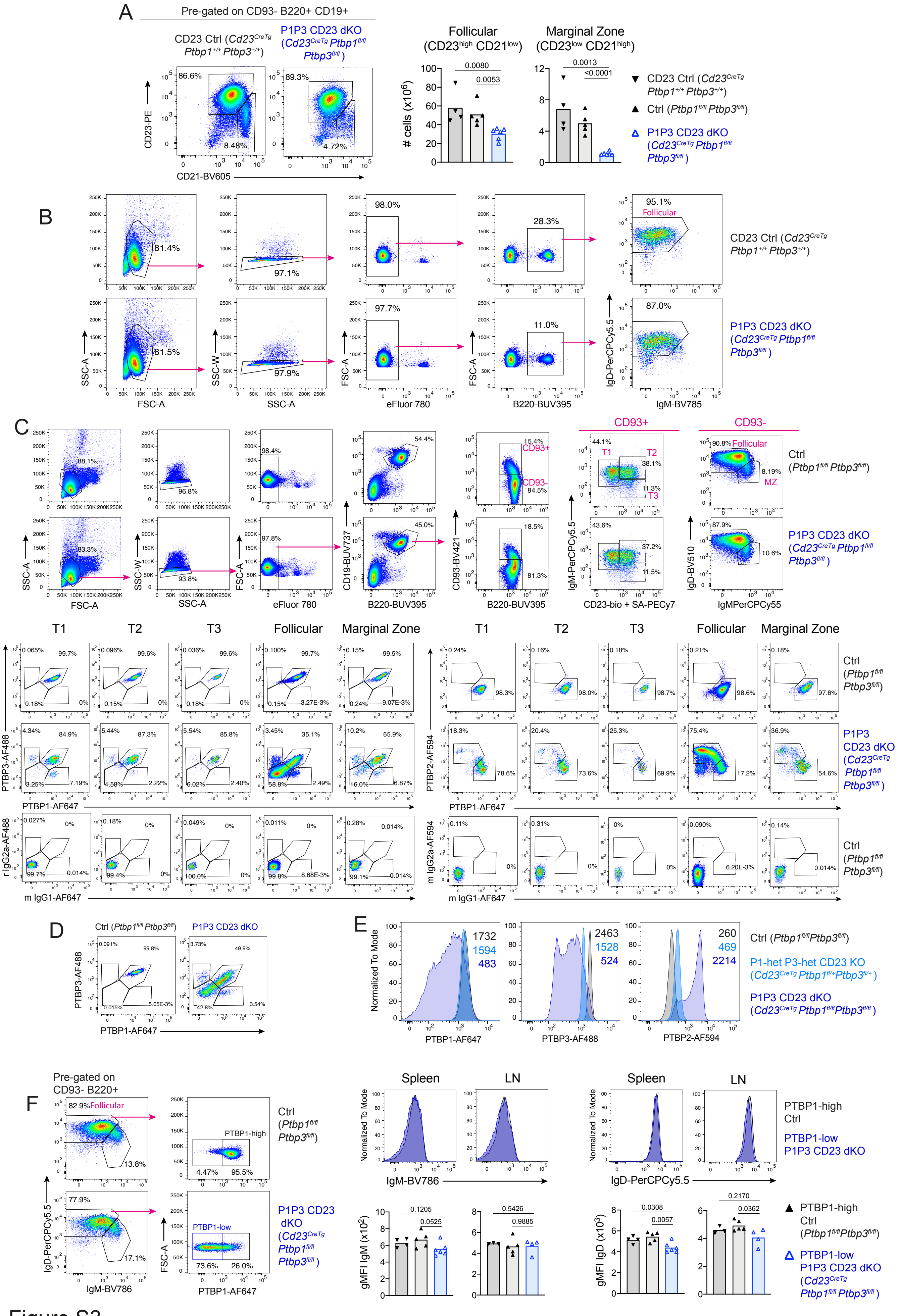

A
Pre-gated on CD93- B220+ CD19+
CD23 Ctrl (Cd23CreTg Ptbp1+/+ Ptbp3+/+)
P1P3 CD23 dKO (Cd23CreTg Ptbp1fl/fl Ptbp3fl/fl)
Follicular (CD23high CD21low)
Marginal Zone (CD23low CD21high)
cells (x10^6)
Ctrl (Ptbp1fl/fl Ptbp3fl/fl)
B
C
CD93+
CD93-
T1
T2
T3
Follicular
Marginal Zone
MZ
D
E
P1-het P3-het CD23 KO (Cd23CreTg Ptbp1fl/+Ptbp3fl/+)
F
Pre-gated on CD93- B220+
Spleen
LN
PTBP1-high Ctrl
PTBP1-low P1P3 CD23 dKO
gMFI IgM (x10^2)
gMFI IgD (x10^3)

Figure S3

Figure S3. PTBP1 and PTBP3 are necessary for the maintenance of mature B cells.
(A) Flow cytometry analysis of splenic B cell populations amongst live (eFluor780-) B220+CD19+CD93- single lymphocytes. Numbers of cells in the spleen identified as shown on the left by flow cytometry. Bars show arithmetic means. Each symbol shows data from one mouse. P values from two-tailed unpaired Student's t-test are shown. Data shown are representative from one out of two independent experiments.
(B) Flow cytometry gating strategy to identify mature B cells in lymph nodes.
(C) Intracellular staining for the different PTBPs and respective control staining using isotype matched antibodies in different populations of splenic B cells analysed by flow cytometry. Top two rows show gating strategy of the different B cell populations analysed.
(D) Intracellular staining for PTBP1 and PTBP3 in B cells (eFluor780- B220+ CD19+) from lymph nodes analysed by flow cytometry.
(E) Intracellular staining for the different PTBPs in B cells (eFluor780- B220+ CD19+) from lymph nodes. Numbers shown in histograms show geometric mean fluorescence intensities (gMFI).
(F) Flow cytometry gating strategy to identify PTBP1-high and PTBP1-low B cells. Histograms show a comparison of IgM and IgD staining between PTBP1-high and PTBP1-low B cells. Graphs show geometric mean fluorescence intensities (gMFI) from IgM and IgD staining. Bars show arithmetic means. Each symbol shows data from one mouse. P values from two-tailed unpaired Student's t-test are shown.

Table S1. Genes bound by PTBP1 and PTBP3

List of genes bound by PTBP1 or PTBP3 anywhere in the transcript (genes) or bound on different features (introns & CDS, 5’-UTR and 3’-UTR).
